# Translation and cultural adaptation of the Minnesota Living with Heart Failure Questionnaire for use in Pakistan

**DOI:** 10.1371/journal.pone.0360245

**Published:** 2026-10-09

**Authors:** Ambreen Gowani, Colleen Norris, Gail Low, Khairulnissa Ajani, Ahmed Noor, Matthias Hoben

**Affiliations:** 1 University of Alberta, Edmonton, Canada; 2 Aga Khan University School of Nursing and Midwifery, Karachi, Pakistan; 3 The Indus Hospital, Opposite Darussalam Society, Karachi, Pakistan; 4 York University, North York, Ontario, Canada; Marwadi University, INDIA

## Abstract

Several tools measure the Health-related quality of life (HRQOL) of people with chronic illnesses but evidence on their cross-cultural adaptation is limited. This study performed the cross-cultural adaptation of the Minnesota Living with Heart Failure Questionnaire (MLHFQ) for use with Urdu speakers in Pakistan, while documenting the adaptation process in detail. This methodological study employed a structured cross-cultural adaptation and linguistic validation design. We incorporated a Delphi Pannel to the MAPI Research Trusts’ 5-step linguistic validation process. Two forward translators independently adapted and reconciled the Indian Urdu version of the MLHFQ for use in Pakistan. Using a modified Delphi approach comprising three Heart Failure patients and one cardiologist reviewed the translation for linguistic accuracy, cultural relevance, and comprehensiveness, identified problems and provided feedback that informed the Pakistani Urdu version 1.0. Two independent back translators translated version 1.0 back to English. The Principal Investigator and back translators reconciled back-translations and proposed changes that were approved by forward translators. Translations, reconciliation reports, and Delphi panel feedback were analyzed using inductive qualitative content analysis, resulting in four themes describing the methodological challenges and adaptation strategies encountered during cross-cultural adaptation: (1) direct transferring of compatible words from the Indian Urdu version to Pakistani Urdu version of the MLHFQ, (2) using indirect substitutes for words lacking direct Urdu equivalents, (3) balancing the tone of written and spoken Urdu, and (4) incorporating contextual nuances within each element of the tool. This study successfully adapted the MLHFQ for Urdu-speaking patients with Heart Failure in Pakistan while systematically documenting the linguistic, cultural, and contextual challenges encountered throughout the adaptation process. The findings offer valuable methodological insights for researchers adapting HRQOL tools and provide quality evidence regarding the use of the MLHFQ for use with Urdu speakers in Pakistan.

## 1 Introduction

Globally, an estimated 56.3–64.3 million people suffer from Heart Failure (HF), making it a significant public health concern due to its progressive nature and the high costs of treatment [1,2]. Over the past two decades, advances in HF treatment have markedly improved chances of survival and Health-Related Quality Of Life (HRQOL) of individuals living with HF [2]. As HRQOL gains recognition as a priority outcome in HF treatment, researchers increasingly require valid and reliable tools to assess the impacts of HF beyond mortality. Of the > 30 measures of HRQOL in HF patients, both generic and disease-specific, nearly all were developed in Western countries and in English, involving HF patients and experts from those regions [3,4]. Despite the significant proportion of global HF patients residing in South Asia [5], no HRQOL instrument has been developed specifically for South Asian populations. In this region, researchers commonly use translated tools initially developed in English-speaking contexts [6–8].

HF profoundly affects an individual’s HRQOL, which, although deeply personal, encompasses universally experienced challenges that surpass cultural boundaries—albeit with varying intensity [9–11]. Studies have identified common issues faced by HF patients worldwide, including limitations in functional capacity, psychological well-being, and social roles [10]. These shared experiences offer a valuable foundation for assessing HRQOL among HF patients using cross-culturally adapted tools.

Cross-cultural adaptation ensures that the meaning of a questionnaire’s instructions, items, and response options is preserved between the source and target cultures [12]. To facilitate meaningful comparisons across cultures, it is essential to establish conceptual, semantic, operational, and psychometric equivalence in the target setting [13]. Conceptual equivalence refers to achieving a similar meaning of the **construct** in both cultures. Semantic equivalence means retaining the meaning of **each item** in both cultures. Operational equivalence refers to a similar **questionnaire format, measurement methods, and mode of administration** in both cultures [14]. Achieving these forms of equivalence is a complex process that requires systematic adaptation and transparent reporting of each methodological step [15]. However, researchers often focus solely on the translation steps without explaining what these steps involve in for the specific cultural context, or they conflate cross-cultural adaptation with validation, reporting more details on validation while only briefly mentioning the adaptation process [16]. International recommendations, including those from COnsensus-based Standards for the selection of health Measurement Instruments (COSMIN), International Society for Pharmacoeconomics and Outcomes Research (ISPOR), and the World Health Organization (WHO), advocate for systematic cross-cultural adaptation and transparent reporting to ensure conceptual equivalence and content validity. Despite these recommendations, no universally accepted reporting guideline exists for cross-cultural adaptation studies [16]. All of this consequently limits researcher from fully assessing the tool’s appropriateness in the target culture.

Although existing cross-cultural adaptation guidelines provide recommendations for achieving semantic and linguistic equivalence between source and target languages, they offer limited guidance on how researchers should resolve translation challenges and select acceptable linguistic alternatives [16,17]. Furthermore, published adaptation studies rarely report the steps and rationale underlying linguistic and cultural adaptation decisions, limiting readers’ ability to evaluate the conceptual appropriateness of an adapted instrument for the target population and critically appraise the adaptation process [15]. It is the thorough reporting of the translation and adaptation methods, along with the challenges encountered and strategies employed, that guides interpretation of the tool’s validity and reliability for the target culture [15,16]. To address these knowledge gaps, the primary objective of this study was to develop a culturally adapted Pakistani Urdu version of the MLHFQ. The secondary objective was to systematically document the linguistic and contextual challenges encountered throughout the adaptation process and the strategies used to address them to transparently report the adaptation process.

## 2 Methods

This study was informed by a constructivist paradigm, which recognizes that individuals interpret questionnaire items within their cultural, linguistic, and social contexts [18]. Accordingly, our cross-cultural adaptation process engaged patients, translators, clinician, and researchers in an iterative process to explore the meaning of each element of the tool and collaboratively finalize culturally appropriate wording. Within this paradigm, conceptual equivalence of the questionnaire is co-constructively achieved through stakeholder engagement rather than as the product of linguistic translation alone [19].

We modified the MAPI Research Trust’s five-step process of linguistic translation for adapting the MLHFQ for use in Pakistan [20,21]. The five steps include forward translation and reconciliation, backward translation and reconciliation, and review of the translation by MAPI Research Trust. To enhance the rigor of the adaptation process, we added a Delphi panel with experts [21] to review the forward translation and identify linguistic inaccuracies before translating the tool to English (Fig 1). The Delphi panel aimed to a) compare and discuss the cultural relevance, translational accuracy, and comprehensiveness of each element of the Pakistani Urdu version 1.0, b) highlight discrepancies and find solutions, and c) review suggestions, compare meanings with their English counterparts, and reach consensus on the most suitable substitutes. Experts included three HF patients and a Cardiologist. The core research team comprised three international nurse researchers and two PhD-scholars living in the target culture – all having experience in translation and cultural adaptation of the tools. The entire process of adaptation was carried out between January 2024 and June 2024.

**Fig 1:**
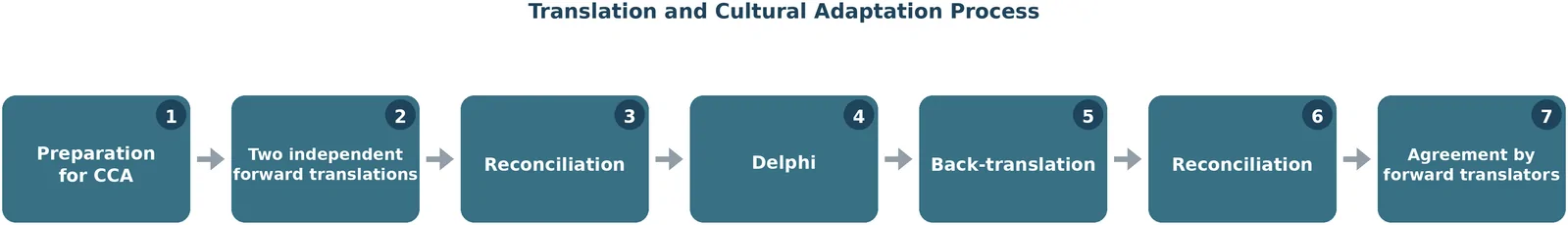
The figure illustrates the sequential process of adapting the Indian Urdu MLHFQ for use with Pakistani patients, including forward translation, reconciliation, back-translation, modified Delphi review, and final reconciliation to ensure linguistic, conceptual, and cultural equivalence.

### 2.1 Preparing for the translation

While searching the MAPI Research Trust’s tool repository, we identified the Indian Urdu Version of the MLHFQ. Considering the cultural and linguistic similarities between India and Pakistan, we decided to adapt the Indian Urdu version of the MLHFQ for use with Urdu speakers in Pakistan. Urdu is a common language widely spoken and understood across both countries. We sought and received permission from MAPI Research Trust to adapt the Indian Urdu version for use in Pakistan.

### 2.2 Ethical considerations

In line with the principles of the Declaration of Helsinki, before executing the study, we obtained ethical approvals from the University of Alberta [Pro00123875] and both study sites [Aga Khan University Hospital Karachi- 2023-8706-26817 and The Indus Hospital- IHHN_IRB_2023_10_018]. All participants provided verbal as well as written informed consent for Delphi and its recording. The PI deidentified the transcript before analysis and secured all forms of data in accordance with the institutional policies.

### 2.3 Forward translation/adaptation and reconciliation

First, two forward translators (AG and KA) independently adapted the four elements (title, instructions, lead-in stem and items, and response choices) of the tool. Both researchers are native Urdu speakers who received their education from English-medium schools, born and brought up in the Pakistani culture, and familiar with the concept of HRQOL. To ensure conceptual, semantic, and operational equivalence between the English, Indian, and the Pakistani versions of the questionnaire, both translators followed a systematic process. They meticulously compared each element of the Indian Urdu version with its English counterpart, assessed similarities in meaning, cultural appropriateness of the wording, item length, and sentence structure. Throughout this process, they identified problematic elements, highlighted potential issues of wordings in the context of Pakistan, and proposed solutions to enhance cultural relevance and bridge the gaps in meaning between the three versions of the MLHFQ.

After producing their respective Pakistani Urdu versions, both researchers met to reconcile their results. Following an item-by-item approach, they simultaneously compared each element of the tool for its meanings in English, Indian Urdu, and Pakistani Urdu, and discussed the challenges faced in adapting each item. They also examined contextual relevance of each item and substituted mutually agreed upon culturally appropriate vocabulary where necessary. The meeting was audio recorded. After the meeting, AG prepared an item-by-item report documenting the key problems, discussion, and decisions taken by mutual consensus to produce a reconciled Pakistani Urdu version 1.0 of the MLHFQ to be considered by the Delphi panel.

### 2.4 Delphi to review the forward adaptation of the MLHFQ

In addition to the PI and a senior HF specialist practicing in Pakistan, we purposively selected three HF patients (two females and one male) who were bilingual in Urdu and English, had been living with HF for at least six months, and were clinically stable enough to participate in a one-hour modified Delphi panel. Purposive sampling ensured that participants integrated their lived experience of HF while evaluating the questionnaire’s language, comprehensiveness, cultural relevance, and acceptability. The PI identified and approached eligible participants during their routine cardiology clinic visits approximately one week before the modified Delphi panel, explained the study’s purpose and procedures, and obtained verbal consent. Patients who agreed to participate were invited to attend the panel at a mutually convenient date and time, with transportation costs (PKR 2,000) reimbursed to all patient participants to facilitate participation. Data collection comprised evaluation forms including the quantitative ratings and written comments by the participants; field notes taken by the PI; and an audio recording of the panel discussion.

On the day of the Delphi, each participant provided written informed consent for participating in the Delphi and its audio recording. The discussion was facilitated by the PI (AG) and conducted in Urdu, with reference to the English version of the MLHFQ where clarification was required. The PI distributed the English and Pakistani Urdu Version 1.0 of the MLHFQ along with an evaluation form and instructed participants to compare each element of the English version with its corresponding Urdu translation. Over approximately 45 minutes, participants independently reviewed the questionnaire and rated the cultural relevance, translational accuracy, and comprehensiveness of each element of the tool on a four-point Likert scale (1 = least relevant, accurate, or comprehensive; 4 = most relevant, accurate, or comprehensive). They also underlined problematic words or phrases and provided written suggestions for improvement.

Following the independent review, participants submitted their completed evaluation forms to AG, who identified all elements rated 1 or 2 in any domain which were subsequently discussed by the panel. Participants shared their concerns, proposed contextually appropriate alternatives, and refined the wording until consensus was reached among all members on the most appropriate phrasing for each element. Discussions addressed the title, preamble, response options, instructions, and all 21 items. The numerical ratings were not intended to establish quantitative content validity but rather to flag elements requiring discussion. As consensus was achieved in the first round, no additional Delphi rounds were conducted. The audio recording was transcribed verbatim, and the transcript, together with field notes, participants completed evaluation forms, and written comments, was used to prepare a detailed item-by-item consensus report documenting the ratings, identified issues, discussions, and final consensus decisions.

### 2.5 Data analysis

The PI conducted the analysis of the Delphi data. The Delphi discussion was audio-recorded and transcribed verbatim, and the transcript was checked against the recording to confirm accuracy and completeness. Both the transcript and recording were used throughout the analysis to understand the context and intent behind participants’ discussions. Each element of the questionnaire including the title, preamble, response scale, and all 21 items was analysed separately. The transcript was reviewed repeatedly alongside the corresponding audio to build familiarity with the data.

The primary document used for analysis was a Microsoft Excel master spreadsheet originally developed during the forward translation reconciliation meetings between the two forward translators. This spreadsheet already contained details of the translation reconciliations, including translators’ reconciled wordings, translational challenges, and their solutions. Combining evidence from both the forward reconciliation and the Delphi review into a single document, the Delphi panel’s ratings, written comments, discussion summaries, and field notes were then added to the corresponding column for each element. This integrated document maintained an audit trail linking each questionnaire element to its adaptation challenge and the decisions made throughout the forward adaptation process. The document, transcript, and audio recording were used together for content analysis.

The content analysis proceeded stepwise. First, elements that had achieved consensus without requiring language changes were identified and retained unchanged. Elements needing minor linguistic or grammatical refinement were revised next. Elements requiring substantial revision were then examined in detail; these mainly involved words or phrases lacking appropriate Urdu equivalents, or items where contextual differences altered the intended meaning.

To consolidate the underlying translation and adaptation issues for each element, notes from the forward reconciliation, Delphi ratings, panel discussion, participants’ written comments, and field notes were compared simultaneously. Each challenge was assigned a descriptive code reflecting the specific issue encountered. Codes were reviewed across all questionnaire elements and compared for similarities and differences. Conceptually similar challenges were then grouped into broader categories to develop a thematic scheme describing the translation and adaptation challenges.

Recognizing that our professional expertise, language proficiency, and familiarity with the target culture, HF and HRQOL could influence the interpretation of participants’ feedback and subsequent translation and adaptation decisions, the preliminary analysis and proposed revisions were discussed with the second forward translator across two data analysis meetings. Through iterative discussion and consensus, a final forward-translated version of the questionnaire was produced. This version was then reviewed by the broader research team before proceeding to the back translation stage. All translation and adaptation decisions were finalized through iterative engagement with the independent translators, clinician, and HF patients [22]. These discussions critically examined interpretations, resolved disagreements, and enhanced the credibility of the analysis.

### 2.6 Back translation of the MLHFQ Pakistani Urdu Version into English

Both back translators are medical students, native Urdu speakers, graduates from Cambridge school systems, and fluent in English. Back translators were blind to the English Version of the MLHFQ and did not discuss their translations with each other. They submitted their respective independent back translations to the AG for further evaluation.

### 2.7 Back translation reconciliation

After receiving the two independent back translations, the PI placed each one next to its corresponding Urdu item in the integrated master document and compared them line by line. Items that matched closely in wording and meaning were marked as agreed. Items that differed were flagged and discussed in a reconciliation meeting attended by the PI and both back translators.

Once the team agreed on the back translations, they compared the reconciled version with the original English MLHFQ to check whether each item still captured its intended meaning. Items that closely matched the original were approved. Instances where meaning had shifted, the team discussed its possible reasons and classified the discrepancy as either minor or major. Minor discrepancies did not require any change to the forward adapted Urdu version, however, major discrepancies, which altered the intended meaning of an item, were discussed in more depth, and alternative wordings were proposed. These revisions were then sent to the second forward translator for review and approval. For concepts that had no direct Urdu equivalent, differences in the back translation were accepted as long as the underlying meaning had been preserved. The reconciled Urdu version was then carried forward to the cognitive debriefing interviews, which are described in a separate manuscript.

### 2.8 Trustworthiness

The study adheres to the Lincoln and Guba’s criteria for trustworthiness through its robust methods and transparent reporting [23]. Credibility and confirmability were enhanced by verifying the Delphi transcript against the audio recording, integrating multiple sources of evidence (forward reconciliation documentation, Delphi ratings, written comments, discussion summaries, and field notes) for analysis and iteratively discussing interpretations with the second translator and the research team until consensus was reached. Dependability was supported through a systematic item-by-item analysis and by maintaining an audit trail documenting adaptation challenges, coding decisions, translation revisions, and questionnaire versions. Transferability is supported through transparent reporting of the adaptation process, challenges, and solutions making the findings applicable to similar settings [23,24].

## 3 Result

### 3.1 Translation and adaptation of the MLHFQ

We identified four major themes: 1) direct transfer of wording from Indian Urdu Version to Pakistani Urdu Version, 2) lack of relevant substitute vocabulary in Urdu, 3) discrepancy in spoken and written Urdu, and 4) contextual variation in the applicability of the items. All the themes are presented in Table 1.

**Table 1:** Examples of Adaptations for each theme.

Theme	English Version	Indian Urdu Version	Pakistani Urdu Version	Approved Adaptations
Direct Transfer	Making your going places away from home difficult	Aap ke ghar se door ki jaghon par jaaney ko mushkil kar ke	Aap ke ghar se door jaghon par jaana mushkil kar ke	The Indian Urdu wording was understandable but unnecessarily long. The phrase was shortened via minor grammatical changes without changing the intended meaning.
Lack of relevant substitute in the target language	Minnesota Living with Heart Failure Questionnaire	Minnesota *“Sikta-e-Qalb*” ke saath Zindagi guzarney ka sawalnama	Minnesota “*Dil ki kamzori”* ke saath zindigi guzarney ka sawalnama	*Sikta-e-Qalb* means cardiac arrest rather than heart failure*. Dil ki kamzori* was selected as a more appropriate expression used in clinical practice.
Discrepancy in spoken and written language	Making you eat less of the foods you like	Aap ko aap ke pasandidah khaaney ko kam khaney par aamadah kar ke	Aap ko majboor kar ke, kea ap apney pasandidah khaney kam khayein	The Indian Urdu sentence structure was complex and less natural in spoken Urdu. We modified the sentence structure and used familiar alternative for “*making*”.
Contextual application	Making your working around the house difficult	Aap ke ghar ke gird ya baagh me kaam karney ko mushkil bana ke	Aap ke ghar ya sehan me kaam karney ko mushkil kar le	“*Baagh*” (garden) was considered less contextually appropriate. “*Sehan*” more appropriately relates to the local housing structure in Pakistan. The sentence was also simplified.

#### 3.1.1 Theme 1: Direct transfer of wording from Indian to Pakistani Urdu.

We transferred items 5 (going away from home), 8 (earn a living), 10 (sexual activities), 12 (shortness of breath), and 19 (worry) from Indian Urdu version to Pakistani Urdu version with minimal or no changes. These items were linguistically appropriate and closely aligned in meaning with their English counterparts. One translator shared:

“*These (items 5 and 10) are simple sentences. They are just fine as it is with same meanings in English and Urdu*”.

They comprised easy, simple, and culturally appropriate words that did not have any ambiguity in Urdu. They consistently carried the same meaning across the three MLHFQ versions throughout forward and backward translations. Although item applicability to Delphi participants varied, items were well understood by all of them. One Delphi participant commented (written);

“*This is ok. No changes required*.”

#### 3.1.2 Theme 2: Lack of relevant substitute vocabulary in Urdu.

Certain words, such as “heart failure,” “well,” “recreational pastimes,” “fatigue,” and “relating to,” lacked direct equivalents in Urdu. The Indian Urdu version used indirect translations, which failed to fully convey their intended meaning. To improve clarity, we replaced these words with Urdu words more closely aligned with their original meanings. While these replacements were not entirely precise, they did convey the intended message more clearly than the previous wording for Delphi participants.

For example, the term “HF” lacks a linguistically precise equivalent in Urdu. Its literal translation implies “cardiac standstill,” “cardiac arrest,” or “death (heart fail),” none of which accurately convey the clinical condition of HF. The Indian Urdu version referred to HF differently at two places within the tool, using “*sikta-e-dil*” and “*dil ka dora*,” meaning “heart’s state of shock” and “heart attack,” respectively. Neither term reflects the concept of HF as intended in the tool. We therefore replaced “HF” with the phrase “*dil ki kamzori*,” meaning “weakness of the heart.” While this term indirectly suggests HF with reduced ejection fraction, it is commonly used by Pakistani physicians to describe reduced and preserved ejection fractions. Translators as well as Delphi participants discussed concerns about the Urdu alternatives for the term “HF” conveying substantially different conditions. For example one of the translators remarked,

*“People live with heart failure for decades. It does not mean cardiac arrest or heart attack. It is a completely different problem. And surprisingly there is no word for it.”*

The inconsistency in the translations was also noted by the Delphi participant. One participant noted;

*“English copy says heart failure, Urdu copy says a weak heart—are they both same?”*

Similarly, the terms “fatigue” and “tired” may seem synonymous, but they carry subtle differences in their definitions in both English and Urdu. The literal translation of both terms in Urdu is “*thakawat*,” which can be back translated as either “tired” or “fatigue.” However, the Indian Urdu version translated “fatigue” as “*shikasta*,” which means “defeated” in English. Conceptually, “defeated” does not equate to fatigue. For clarity, we used the single term “*thakawa*t” to represent both “tired” and “fatigue.” This word choice effectively preserved the intended meaning while reducing the length of the item. One translator explained why “*Shikasta”* was an in appropriate Urdu alternative:

“*Shikasta is different from tiredness. It hints towards mentally drained and a defeated person.*

#### 3.1.3 Theme 3: Written vs spoken Urdu.

During the forward adaptation process, we recognized that the Indian Urdu version of the MLHFQ was linguistically sophisticated and formal, making it less relatable and engaging for the Urdu-speaking population in Pakistan. It contained words more commonly found in written Urdu than in spoken Urdu, which could hinder comprehension. Hence both forward translators carefully examined each component of the tool for colloquial appropriateness in Urdu. There is a significant difference between everyday spoken and written Urdu. Written Urdu tends to be dense and highly formal, typically used in contexts such as literature, poetry, legal documents, and journalism. In contrast, spoken Urdu features simpler vocabulary, easier pronunciation, and a casual tone for persons with varying levels of literacy. Two key adaptations are discussed below.

During the adaptation process, another important challenge was to translate and to fit the word “making” in context. In Urdu, the dictionary definition of “making” refers to physically creating, building, or developing something. In the MLHFQ, it is used to accentuate the extent to which HF patients are affected by their condition on various aspects of daily living. Unlike English, the formal Urdu translation of “making” does not fit well with all activities, so we needed to find relevant colloquial alternatives. For example, for item 11, we replaced “making” with the Urdu term “*majboor*,” which translates to “compelling.” HF compels people to eat foods that they may dislike. For example, one translator explained why making could not be substituted by a single Urdu term.

*“I could not translate making consistently for all items. I had to use different terms like forcing, causing, compelling, for different items to keep the meaning intact.”*

Likewise, the Indian Urdu version translates “previous” as “*Guzishta*,” a formal term to refer to the past. We opted for the simpler and more commonly used word “*peechlay*” which, when back translated, means “previous” or “past.” Also, the Indian Urdu version used the term “*Daryaft*” for the term “ask”, a formal and less commonly used term. We replaced it with “*poochtay*,” which conveys the same meaning and more casually engages responders. For example, a Delphi participant asked:

*“What does this [guzishta] word mean?”*

#### 3.1.4 Theme 4. Contextual nuances.

While most items in the tool address general limitations in daily activities, some were less applicable to the Pakistani context. For example, item 2 asks how much HF bothers a patient by compelling them to rest during the day. In Pakistani culture, afternoon naps are common and may not necessarily be a result of HF. Thus, it was essential to differentiate habitual daytime rest from sitting or lying down that is potentially imposed by HF. Both forward translators agreed on using verbs that convey a sense of compulsion or compromise. We replaced “making” with “*majboor,*” which means “compelling.” Although this choice does not yield an exact back translation, all translators and Delphi participants approved it as culturally appropriate and closest in its intended meaning. Discussing the translations for item 2, one of the forward translators suggested:

*“For example, my mother takes an afternoon nap every day, whether she is sick or not. If you ask her this question, how would she know whether she is resting because of her usual routine or because of her disease?”*

Item 4 assesses the impact of HF on activities related to working around the house or yard. The Indian Urdu version had translated “yard” as “*baagh*” meaning garden, which is both linguistically and contextually inaccurate. Most Pakistanis live in flats with balconies or small outdoor areas, so using “garden” could lead to confusion. Given the differences in housing structures between the USA and Pakistan, we translated “yard” as “*sehan*” – a more inclusive term that refers to any type of outdoor space around a house. This adjustment improved clarity and ensured participants provided more accurate responses.

While explaining the absent rating on the review document, one of the Delphi participants shared:

*“I live in a flat. There is no garden in my house. So this question is not applicable for me.”*

## 4 Discussion

Adapting an instrument for a new culture requires substantial time, effort, and resources to ensure equivalence between source and target versions of the questionnaire. However, the process is rarely documented in enough detail to establish an instrument’s trustworthiness in the target culture. This study addresses that gap by providing a detailed account of adapting the MLHFQ for Urdu-speaking population in Pakistan, accounting for the linguistic, geographic, cultural, and infrastructural (e.g., housing-related) differences between the American (source) and Pakistani (target) contexts. We describe the challenges and solutions behind key translation choices and the lessons learned throughout the process. The findings provide future researchers a clear basis for using the MLHFQ in Pakistani culture.

One of the greatest challenges encountered during adaptation was translating the term “heart failure,” a central concept of the tool. No direct Urdu equivalent fully captures the clinical meaning of HF. The difficulty lies primarily in the term “failure,” which can imply an end, collapse, hopelessness, or catastrophe and may be perceived as far more threatening than the actual clinical syndrome [25,26], This challenge is not unique to Urdu; even physicians in English-speaking cultures have expressed hesitation in using the term with patients and have proposed “cardiac impairment” as a broader and less alarming alternative [26].

We used a colloquial Urdu term commonly used by Pakistani physicians to describe HF. Although the Delphi panel endorsed this wording, it is not a precise semantic equivalent of “heart failure.” Previous studies have also reported using proxy terms in cross-cultural adaptation when direct target-language equivalents are unavailable [15]. However, a culturally familiar and understandable term may not fully preserve the meaning of the original construct. Therefore, translation choices should be explicitly reported, including the rationale for their selection, cultural applicability, and potential implications for interpreting the adapted tool. Such detailed reporting remains limited in cross-cultural adaptation studies, and existing translation guidelines provide little guidance on how these translation choices should be selected and documented [17]. Our Delphi process helped identify and establish consensus on the most appropriate alternatives for the Pakistani context. We recommend that MAPI guidelines provide clearer guidance for such situations, particularly on documenting alternative wordings, the rationale for their selection, and their potential implications for the meaning and interpretation of the tool in the target culture [27–29].

Given the low literacy rate among Pakistanis aged 65 and older, it was crucial to simplify the language of our tool to ensure that it was comprehensible and engaging for the target population. Guidelines as well as previous researches have also highlighted the risks associated with using unfamiliar terminology and suggest translating the tool using language commonly spoken by the population [30]. Striking a balance between formal and colloquial Urdu was challenging [31]. Fortunately, both translators were familiar with the nuances of written and spoken Urdu and were able to identify appropriate colloquial alternatives. While the Indian Urdu version was linguistically sound, it was dense and less natural in spoken Urdu and, therefore, less culturally appropriate for our target population. It was even less engaging and more difficult to understand for persons with low literacy. It could reduce comprehension and potentially affect responses to sensitive items related to sexual activities, psychological distress, and social support Some languages have significantly different written and spoken forms, necessitating greater effort to adapt tools for clarity and accessibility for all users [30,32]. Weighing different word choices against the population’s literacy levels, anticipating their potential consequences, selecting an appropriate one and then clearly documenting the process adds authenticity and credibility to the adapted version and provides potential readers and users a clear basis for how the tool might function in the target culture and what could be its potential limitations. and Current guidelines also suggest using the language that is easy and locally understood, they do not mandate researchers to weigh the alternatives and report the caveats of using one over other. We recommend raising researchers’ awareness about this kind of discrepancy and the process of resolution in cultural adaptation guidelines.

In this study, engaging a Delphi panel of cardiologists and HF patients was an essential quality control step between forward and backward translation [33,34]. There were open discussions and focused feedback from an end user perspective to improve accuracy, comprehensiveness, relevance, and clarity in forward translations [34]. While guidelines for translation and cultural adaptation of research tools do not typically include a Delphi phase [20], it saved time, enhanced the language of the tool, and provided a solid foundation for translational decisions. We highly recommend incorporating the Delphi method into translation guidelines to improve translation quality and process reporting.

Our adaptation process duly considered cultural and contextual differences between Pakistani and American society, and adaptations based on input from Pakistani persons living with HF. Two key cultural differences warrant discussion: a) housing structure, and b) practice of daily afternoon naps. Most of the Pakistani population resides in flats with balconies or galleries, while backyards in American homes are typically large, multipurpose outdoor spaces. Pakistani balconies are smaller, indoor areas with limited functionality. Working around a yard involves greater physical endurance than working around a balcony. We translated “yard” into Urdu but also included “balcony” as an option—a term befitting of prevalent housing types in Pakistan. This adaptation made the wording of this item more understandable and relatable.

Similarly, afternoon naps are a common practice among older adults in Pakistan. Participants might be taking daytime naps as part of their regular routine, regardless of their HF, which could lead to inflate item scores. To differentiate between routine sleeping and exhaustion, we opted for a more compelling verb that better clarified the intent of the item. These cultural differences necessitate significant and valid modifications to MLHFQ, which should be documented to help researchers grasp the nuances involved [35,36]. Current translation and adaptation guidelines do not adequately assist translators in addressing these challenges [30]. Selecting appropriate wording, recognizing cultural differences, and fine-tuning language are critical aspects of tool adaptation that existing methodological guidelines do not adequately address [30,37,38]. This study makes important recommendations for change, for additional criteria and instructions to be included in these guidelines.

### 4.1 Strengths and limitations

The study employed a structured methodology for the cross-cultural adaptation of the MLHFQ, including a modified Delphi to ensure the quality of the adaptation process. Our purposive sampling and sample size were appropriate for capturing cultural diversity rather than statistical representation [39]. Nonetheless the small sample, single-country setting, and focus on one language limit the transferability of findings to Urdu-speaking populations outside Pakistan and to Pakistanis who speak other regional languages. Also, only one clinician participated in the adaptation process, which may have restricted other clinical perspectives informing the adaptation decisions. Moreover, we reached consensus through a single modified Delphi round which limited the opportunity for further iterations. Cognitive interviews are reported separately in a subsequent manuscript; therefore, response process validity and patients’ interpretation of the adapted items are beyond the scope of the present study. Finally, this study focused on translation and cultural adaptation only; it did not assess psychometric properties such as reliability and validity. These require evaluation in subsequent studies of the adapted MLHFQ among people living with HF in Pakistan.

## 5 Conclusion

Cross-cultural adaptation methodologies have significant gaps, lack consensus, and provide limited guidance on how researchers can address differences between source and target languages and cultures. While cross-cultural adaptation is a prerequisite for evaluating the validity and reliability of tools, inadequate reporting of the adaptation process leaves researchers with limited understanding about how an instrument is expected to perform in a new cultural context. This study highlights the methodological intricacies of cross-cultural adaptation and demonstrates the importance of making translation decisions, their rationale, and their cultural implications explicit. Our findings contribute to strengthening the methodological and reporting processes for cross-cultural adaptation and provide practical considerations for adapting the MLHFQ and other HRQOL instruments to the Pakistani context.

## Abbreviations

HF: heart failure
HRQOL: health related quality of life
MLHFQ: minnesota living with heart failure questionnaire
PhD: doctor of philosophy
PI: principal investigator.
